# Incidence of hyperoxia in trauma patients receiving pre-hospital emergency anaesthesia: results of a 5-year retrospective analysis

**DOI:** 10.1186/s13049-021-00951-w

**Published:** 2021-09-10

**Authors:** P. Leitch, A. L. Hudson, J. E. Griggs, R. Stolmeijer, R. M. Lyon, E. ter Avest, Air Ambulance Kent Surrey Sussex

**Affiliations:** 1 University of St Georges, Tooting, London, UK; 2 Air Ambulance Kent Surrey and Sussex, Hanger 10 Redhill Aerodrome, Redhill, RH1 5YP UK; 3 University of Surrey, Guildford, UK; 4 Department of Emergency Medicine, University Medical Center Groningen, Groningen, The Netherlands

**Keywords:** Hyperoxia, Hyperoxemia, Ventilation, Trauma, Emergency medical services, Oxygen therapy, Pre-hospital anesthesia

## Abstract

**Background:**

Previous studies have demonstrated an association between hyperoxia and increased mortality in various patient groups. Critically unwell and injured patients are routinely given high concentration oxygen in the pre-hospital phase of care. We aim to investigate the incidence of hyperoxia in major trauma patients receiving pre-hospital emergency anesthesia (PHEA) in the pre-hospital setting and determine factors that may help guide clinicians with pre-hospital oxygen administration in these patients.

**Methods:**

A retrospective cohort study was performed of all patients who received PHEA by a single helicopter emergency medical service (HEMS) between 1 October 2014 and 1 May 2019 and who were subsequently transferred to one major trauma centre (MTC). Patient and treatment factors were collected from the electronic patient records of the HEMS service and the MTC. Hyperoxia was defined as a PaO_2_ > 16 kPA on the first arterial blood gas analysis upon arrival in the MTC.

**Results:**

On arrival in the MTC, the majority of the patients (90/147, 61.2%) had severe hyperoxia, whereas 30 patients (20.4%) had mild hyperoxia and 26 patients (19.7%) had normoxia. Only 1 patient (0.7%) had hypoxia. The median PaO_2_ on the first arterial blood gas analysis (ABGA) after HEMS handover was 36.7 [IQR 18.5–52.2] kPa, with a range of 7.0–86.0 kPa. SpO_2_ pulse oximetry readings before handover were independently associated with the presence of hyperoxia. An SpO_2_ ≥ 97% was associated with a significantly increased odds of hyperoxia (OR 3.99 [1.58–10.08]), and had a sensitivity of 86.7% [79.1–92.4], a specificity of 37.9% [20.7–57.8], a positive predictive value of 84.5% [70.2–87.9] and a negative predictive value of 42.3% [27.4–58.7] for the presence of hyperoxemia.

**Conclusion:**

Trauma patients who have undergone PHEA often have profound hyperoxemia upon arrival at hospital. In the pre-hospital setting, where arterial blood gas analysis is not readily available a titrated approach to oxygen therapy should be considered to reduce the incidence of potentially harmful tissue hyperoxia.

## Background

Major trauma results in 20,000 cases in England each year [1]. Helicopter emergency medical services (HEMS) are frequently dispatched to patients after major trauma, since they can deliver specific, advanced clinical interventions that land ambulance crews are unable to provide. Examples of these include thoracostomies to relieve a tension pneumothorax, the transfusion of blood products to treat ongoing blood loss, and pre-hospital emergency anesthesia (PHEA) to definitively manage the airway or to optimise ventilation. To prevent hypoxia during PHEA, patients are normally pre-oxygenated with a high FiO_2_ and oxygen administration is continued thereafter [2, 3].

Oxygen therapy, however, can result in arterial oxygen levels well in excess of the normal maximum physiological levels, causing excessive formation of reactive oxygen species (ROS) and oxygen free radicals which can cause cellular damage [4]. Furthermore, as oxygen is a vasoactive substance, its administration may have unintended haemodynamic effects, such as coronary and systemic vasoconstriction [5–7]. Previous studies have demonstrated an association between hyperoxia and increased mortality in patients following cardiac arrest, sepsis, stroke and traumatic brain injury (TBI) [8–11].

Pre-hospital oxygen administration to trauma patients is therefore a delicate balance and both hypoxia and hyperoxia should be avoided [12]. Where in-hospital oxygen supplementation is normally guided by arterial blood gas analysis (ABGA), suitable tools to guide oxygen therapy in the pre-hospital setting are largely lacking. As many trauma patients receiving PHEA have injuries making them either more prone to hypoxia, or making them more vulnerable to the consequences of hypoxia, clinicians usually tend to prioritize preventing hypoxia over hyperoxia [2].

It remains unclear how many trauma patients receiving PHEA are exposed to prolonged hyperoxia in the pre-hospital setting. Therefore, the aim of this study was twofold: (1) to investigate the incidence of hyperoxia in trauma patients receiving PHEA in the pre-hospital setting, and (2) to determine factors that may help guide clinicians with pre-hospital oxygen administration in these patients.

## Methods

### Study setting and design

This is a retrospective study of all patients who received PHEA from Air Ambulance Kent Surrey Sussex (AAKSS) between 1 October 2014 and 1 May 2019 and who were subsequently transferred to St George’s Hospital (STGH), London. STGH is a designated MTC, providing specialist tertiary care for the South West London and Surrey Trauma Network (SWLSTN), covering a population of approximately 2.6 million. STGH has an on-site helipad and approximately 83% of patients arriving by the HEMS team are flown in. AAKSS is a HEMS provider, covering three counties in the southeast of England with a resident population of 4.5 million and transient population of up to 8 million. Two doctor/paramedic teams respond in either a helicopter or response car from one base. The service attends approximately 2000 patients per year. Most patients attended by the HEMS service are first seen by a ground ambulance crew and/or a critical care paramedic.

PHEA was carried out according to the AAKSS standard operating procedure (SOP). All patients were pre-oxygenated with a high FiO_2_ before rapid sequence induction (RSI). During the study period this was achieved by applying a non-rebreathe mask with 15 L/min of oxygen (O_2_) for several minutes prior to intubation. When a difficult airway was anticipated, additional apnoeic oxygenation (ApOx) was provided by administering O_2_ at 15L/min via nasal prongs during the apnoeic phase of intubation. After RSI, intermittent positive pressure ventilation (IPPV) was initiated using a transport ventilator (either Pneupac ParaPAC 310 or Dräger Oxylog 3000) until handover in hospital. During the study period there was no SOP for reducing FiO_2_ based on high oxygen saturations.

A patient deemed to have life-threatening major traumatic haemorrhage is declared ‘Code Red’ at the discretion of the attending HEMS clinicians. Transfusion therapy consisted of packed red blood cells (PRBC) and freeze-dried plasma (FDP).

### Study population

Patients were deemed eligible if they were 18 years or older, had sustained traumatic injuries, underwent PHEA, and were subsequently transferred to STGH by AAKSS. The initial ABGA had to be performed within 60-min after arrival in STGH. Excluded patients were those < 18 years, patients who underwent PHEA for medical (i.e. non-traumatic) reasons and patients wherein the first ABGA was performed > 60 min following admission (to reduce the risk of confounding by in-hospital treatments).

### Data acquisition

The patient population was selected from the AAKSS electronic database (HEMSBase 2.0 Medic One Systems Ltd, UK) using the following criteria: traumatic injuries, completion of pre-hospital PHEA, HEMS as transport mode and STGH as destination hospital. Pre-hospital data abstracted from HEMSBase included: age, gender, mechanism of injury, body regions injured, initial Glasgow Coma Score (GCS), PHEA pre-oxygenation- and apnoeic oxygenation, the occurrence of oxygen desaturations (SpO_2_ < 90%) peri- or post PHEA, FiO_2_ post intubation, time from PHEA to presentation in hospital and SpO_2_ as measured by pulse oximetry just before handover in hospital.

In-hospital data were obtained from the STGH electronic patient records, and included the first ABGA result in hospital after handover in the Emergency Department (ED). ABGA results were obtained using AQURE, a middleware system used to record and retrieve data from point of care devices.

### Clinical endpoints

The primary endpoint of interest in this study was the incidence of hyperoxemia in the first ABGA performed after handover in the ED. Secondary endpoint of interest was the association of pre-hospital patient- and treatment characteristics with the occurrence of hyperoxemia.

For the purpose of this study, clinically relevant hypoxemia was defined as PaO_2_ < 8 kPa, normoxemia as PaO_2_ 8–16 kPa, mild hyperoxemia as PaO_2_ > 16–26.6 kPa, and severe hyperoxemia as > 26.6 kPa [13, 14].

### Ethical considerations

This project met National Institute for Healthcare Research (NIHR, UK) criteria for service evaluation and formal ethical approval was therefore not required. The project was approved by the AAKSS Research & Development Committee. A data sharing agreement is in place between AAKSS and STGH.

### Statistical analysis

Descriptive statistics are given as mean [95% CI] or median [IQR]. Comparisons across groups (hypoxemia, normoxemia, mild hyperoxemia and severe hyperoxemia) were made using Fisher exact and Kruskal–Wallis tests, where appropriate. Univariate correlation analysis with calculation of Spearman correlation coefficients was performed to evaluate the association of clinical- and treatment factors with the primary outcome. Multivariable logistic regression analyses were carried out to determine which factors were independently associated with hyperoxemia upon arrival in the major trauma center (MTC). Odds ratios (OR) were calculated for these factors. Missing values are reported in the results section of the manuscript according to the STROBE guideline [15]. A *p*-value < 0.05 was regarded as statistically significant. All statistical analyses were conducted using SPSS 27.0 for Mac statistical package.

## Results

### Patient characteristics

**Fig. 1:**
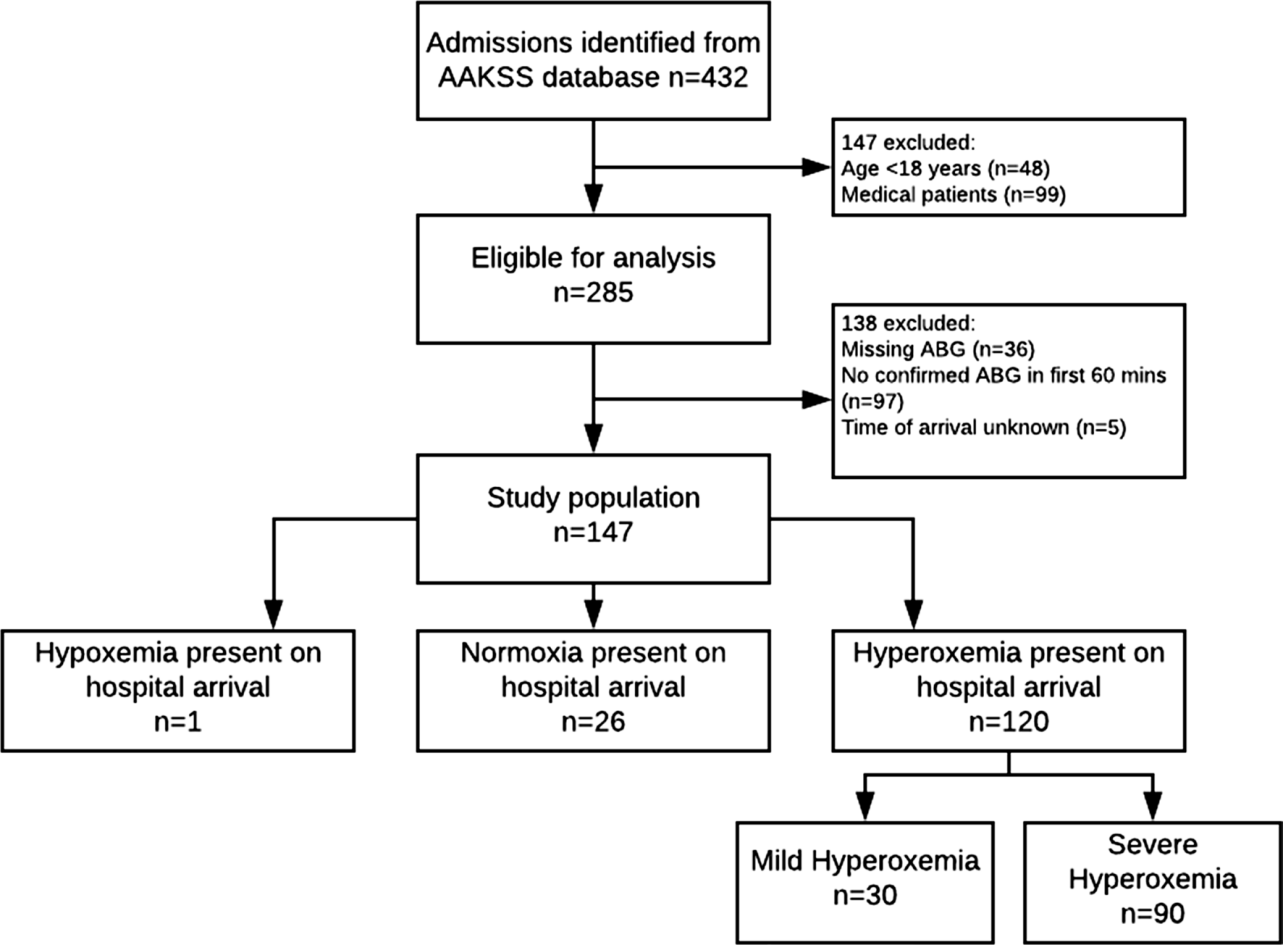
Derivation of study population. Legend: ABGA, arterial blood gas analysis

PHEA was performed in 1241 patients during the study period, 432 patients were subsequently admitted to STGH. 285 fulfilled the inclusion criteria. 76 patients were excluded (5 patients were missing arrival times, 36 had missing ABGA results, and 35 patients had missing time of first ABGA). Further results refer to the remaining 147 patients (Fig. 1).

Patient characteristics of the study population are depicted in Table 1. Most patients were male (77%) and involved in road traffic collisions (RTCs) (46%). Median GCS at presentation was 8, and a significant proportion of the patients had confirmed TBI upon subsequent investigation at the MTC. 25% had chest injuries on presentation and 23% had pre-hospital thoracostomies in addition to PHEA. 17% were declared ‘Code Red’ pre-hospital.

**Table 1:** Patient characteristics of the study population stratified by PaO_2_ in first arterial blood gas analysis after arrival in hospital (n = 147)

	ALL (n = 147)	Hypoxemia (n = 1)	Normoxemia (n = 26)	Mild Hyperoxemia (n = 30)	Severe Hyperoxemia (n = 90)	*p*
*Demographics*						
Age (years, [IQR])	49 [30–65]	29 [NA]	44 [29–53]	53 [29–68]	49 [30–66]	.59
Male, gender [n,%]	116 [78.9]	0[0]	23 [88.4]	25 [83.3]	68 [75.5]	.21
*Mechanism*						
RTC [n,%]	66 [44.9]	1 [100]	12 [46.2]	16 [53.3]	37 [41.1]	.45
Falls [n,%]	54 [36.7]	0 [0]	9 [34.6]	9 [30.0]	36 [40.0]	
Assault [n,%]	10 [6.8]	0 [0]	3 [11.5]	3 [10]	4 [4.4]	
Intentional self-harm [n,%]	9 [6.1]	0 [0]	1 [3.8]	2 [6.7]	6 [6.6]	
Accidental injury (other) [n,%]	3 [2.0]	0 [0]	1 [3.8]	0 [0]	2 [2.2]	
Fire exposure [n,%]	3 [2.0]	0 [0]	0 [0]	0 [0]	3 [3.3]	
Other	2 [1.4]	0 [NA]	0 [NA]	0 [NA]	2 [2.2]	
*Body regions injured*						
Head [n,%]	118 [80.3]	1 [100]	23 [88.5]	22 [73.3]	72 [80]	.52
Chest [n,%]	38 [25.9]	0 [0]	10 [38.5]	11 [36.7]	17 [18.9]	.09
*Vital signs on arrival HEMS*						
GCS [IQR]	8 [4–11]	4 [NA]	5 [3–9]	9 [5–12]	8 [5–11]	.02
SpO_2_ (%, lQR]	98 [88–100]	98 [NA]	92 [88–99]	98 [98–99]	98 [88–100]	.63
SBP (mean, 95%CI)	132 [126–138]	86 [NA]	129 [115–142]	134 [117–150]	133 [126–140]	.005
HR (mean, 95%CI)	96 [91–101]	122 [NA]	104 [92–117]	97 [84–110]	93 [86–99]	.68
*Pre-hospital interventions*						
PHEA						
Pre-oxygenation [n,%]	147	1 [100]	26 [100]	30 [100]	90 [100]	NA
ApOx [n,%]	2	0 [NA]	1 [3.8]	1 [3.3]	0 [0]	.35
Peri-Post-PHEA	56	0 [NA]	15 [57.7]	14 [46.7]	27 [30.0]	.04
Desaturation [n,%]						
Post intubation FiO_2_						
1.0 [n,%]	144 [97.3]	1 [0.7]	26 [34.6]	28 [30]	89 [28.9]	.52
0.5 [n,%]	3 [1.7]	0 [0]	0 [0]	2 [6.7]	1 [1.1]	
Thoracostomies [n,%]*	35 [23.8]	0 [NA]	8 [30.8]	11 [36.7]	16 [17.8]	.14
Transfusion and/or Code Red [n,%]	26 [17.7]	0 [NA]	6 [23.1]	8 [26.7]	12 [13.3]	.31
*In-hospital oxygen treatment*						
Pulse oximetry SpO_2_ HEMS upon arrival in hospital (n = 142)	100 [98–100]	76 [NA]	98 [95–100]	100 [98–100]	100 [99–100]	.01
FiO_2_						
Maintained at 1.0 [n,%]	26 [17.7]	0 [0]	6 [23.1]	2 [6.7]	18 [20]	.82
Downtitrated (0.8–0.2)	120 [81.6]	1 [100]	20 [66.9]	28 [93.3]	71 [78.9]	
Unknown	1	0	0	0	1 [1.1]	
ABGA results						
PaO_2_ [median, IQR]	36.7 [18.5–52.2]	7.9 [NA]	13.8 [11.1–14.9]	19.5 [18.4–23.6]	46.3 [39.0–60.8]	< .001
PaCO_2_ [median, IQR]	6.1 [5.6–7.0]	9.0 [NA]	6.8 [5.9–7.5]	6.3 [5.8–7.1]	5.9 [5.3–6.5]	.016
pH [median, IQR]	7.3 [7.2–7.4]	7.14 [NA]	7.3 [7.2–7.4]	7.3 [7.2–7.3]	7.3 [7.3–7.4]	.002

RTC, road traffic collision; SpO_2_, Oxygen saturation; GCS, Glasgow coma score; SBP, systolic blood pressure; HR, heart rate; PHEA, pre-hospital emergency anesthetic; FiO_2_, fraction of inspired oxygen; ABGA, Arterial Blood Gas Analysis; PaO_2_, Partial pressure of oxygen, PaCO_2_, Partial pressure of CO_2._ *unilateral or bilateral

### Pre-hospital oxygen administration and monitoring

Oxygen was administered with an FiO_2_ of 1.0 in all 147 patients after PHEA and during transport, and only adjusted to FiO_2_ of 0.5 in 3 patients (2%). Pulse oximetry recordings on the HEMS monitor at the moment of handover in the trauma center were available for 142/147 patients. The median [IQR] SpO_2_ at handover was 100 [98–100%]. There were 112 patients (80%) with an SpO_2_ > 98%, 8 patients with an SpO_2_ reading of 95–98%, and 22 patients with an SpO_2_ reading < 95%. Information on the quality of the trace was not available. Following arrival in the ED, 27 patients (18%) were maintained on FiO_2_ 1.0, whereas oxygen was down-titrated in the remaining patients.

### ABGA results

Median [IQR] (95% CI) PaO_2_ in the first ABGA after HEMS handover was 36.7 [18.5–52.2] kPa, with a range of 7.0–86.0 kPa. The majority of the patients (90/147, 61.2%) had severe hyperoxemia, whereas 30 patients (20.4%) had mild hyperoxemia, 26 patients (19.7%) had normoxemia and only 1 patient (0.7%) had hypoxemia. Patient and treatment characteristics stratified by PaO_2_ are represented in Table 1. Patients with (severe) hyperoxemia in their ABGA generally presented to the HEMS team with a higher median GCS before they were intubated, they had less often an oxygen desaturation peri- or post intubation, and had a higher pulse oximetry SpO_2_ on the HEMS monitor at handover in hospital.

### Association of patient characteristics and treatment variables with PaO_2_

Univariate correlation coefficients of PaO_2_ with pre-hospital patient- and treatment variables are represented in Table 2. SpO_2_ pulse oximetry readings on the HEMS monitor at handover in hospital showed a positive association with PaO_2_ (r = 0.28, *p* < 0.001), whilst the occurrence of an oxygen desaturation peri- or post PHEA (r = − 0.23, *p* = 0.006) chest injuries (r = − 0.22, *p* = 0.008) and the treatment of the patient with thoracostomies (r = − 0.19, *p* = 0.022) in the pre-hospital setting were all negatively associated with the occurrence of hyperoxemia.

**Table 2:** Univariate correlation analysis of in-hospital PaO_2_ with pre-hospital patient- and treatment factors (n = 147)

Pre-hospital patient and/or treatment factor	r	*p*
Age	.013	.87
Sex	.052	.53
MOI	.023	.78
Chest injuries	− .220	.008
Head and/or CNS injuries	− .003	.97
Transfusion and/or ‘Code Red’	− .146	.08
Any oxygen desaturation peri-or post PHEA	− .228	.006
ApOx	− .134	0.11
Thoracostomies*	− .190	.022
HEMS Pulse oximetry SpO_2_ at handover in hospital	.276	< .001

MOI; Mechanism of Injury, CNS; central nervous system, PHEA; Pre-hospital Emergency Anesthesia, ApOx; Apnoeic oxygenation, SpO_2_; Oxygen saturation.*unilateral or bilateral

In multivariate logistic regression analysis, HEMS pulse oximetry SpO_2_ at handover in hospital remained the only factor independently associated with the presence of hyperoxemia in the ABGA. Odds ratios for the presence of hyperoxemia were dependent on the SpO_2_ cut-off used (Table 3), but any SpO_2_ ≥ 97% was associated with a significantly increased odds of hyperoxia. In total, 113/142 (79.5%) patients with SpO_2_ values available presented with an SpO_2_ ≥ 97%. An SpO_2_ ≥ 97% had a sensitivity of 86.7 [79.1–92.4]%, a specificity of 37.9 [20.7–57.8]%, a positive predictive value of 84.5 [70.2–87.9]% and a negative predictive value of 42.3 [27.4–58.7]% for the presence of hyperoxemia.

**Table 3:** Multivariate analysis of treatment variables associated with PaO_2_

Variable	OR	95% CI	*p*
PH episode of oxygen desaturation	0.96	[0.89–1.07]	.18
Chest injuries present	0.52	[0.21–1.26]	.29
Thoracostomies*	0.69	[0.27–1.74]	.11
SpO2 at handover ≥ 93%	1.73	[0.56–5.34]	.35
SpO2 at handover ≥ 95%	2.02	[0.69–5.83]	.25
SpO2 at handover ≥ 97%	3.99	[1.58–10.08]	.001
SpO2 at handover ≥ 99%	4.49	[1.85–10.94]	< .001

PH; pre-hospital, SpO_2_; Oxygen saturation.*Unilateral or bilateral

## Discussion

In this study, we demonstrate that trauma patients who have undergone PHEA often have a profound hyperoxemia upon arrival in hospital (61–81%). In the pre-hospital setting, where ABGA is not readily available, a maximum SpO_2_ not higher than 97% may be used as a reasonable goal for FiO_2_ titration to reduce the chance of (deleterious effects of) tissue hyperoxia [4].

Over the past years, the potential deleterious effects of hyperoxemia have become well known. Oxygen levels well in excess of the maximal physiological levels cause excessive formation of ROS resulting in cellular damage [16]. There is compelling evidence that patients exposed to hyperoxia have an increased risk of both short- and long-term mortality [17, 18]. This risk is related to both the extent and the duration of hyperoxia patients were exposed to, although recent in-vivo studies have demonstrated that even a short exposure to supra-physiological oxygen levels may result in haemodynamic changes (reduction in cardiac output and an increase in vascular resistance for example) that may contribute to a worse outcome [19].

Prevention of hyperoxemia in trauma patients after PHEA is of the utmost importance, even more so when transport distances to definitive care are long, such as in our population where the average time from PHEA to presentation in hospital was 59 min. However, several factors may prevent early titration of FiO_2_ by HEMS teams. First, many trauma patients have chest- and central nervous system (CNS) pathology making them both more prone and vulnerable to hypoxic episodes [20]. Most physicians will prefer a period of hyperoxia in these patients above a potential hypoxic episode, and will therefore be reluctant to down-titrate FiO_2_ at an early stage. Second, effective down-titration may be prevented by the settings of (especially older model) transport ventilators who only have a limited range of FiO_2_ options. Further, the lack of a clear target for FiO_2_ titration in the pre-hospital setting may make critical care teams apprehensive to down-titrate FiO_2._ In-hospital titration is normally done based on PaO_2_ results from ABGA. Although pre-hospital blood gas analysis has been shown to be feasible [21] and been demonstrated to have the potential to increase pre-hospital diagnostic accuracy [22], there are limitations to its use (e.g. narrow temperature range of operation) and its use is not widespread across critical care services.

In this study, we demonstrate that in the pre-hospital setting, where ABGA is not readily available, a maximum SpO_2_ not higher than 97% can be used as a reasonable goal for FiO_2_ titration to reduce the chance of (deleterious effects of) tissue hyperoxia in intubated trauma patients. These findings are in-line with the recommendations of the British Thoracic Society Guidelines on use of oxygen in emergency settings, advising that oxygen should be administered to reach a target oxygen saturation range of 94–98% for most critically ill patients [4]. Titration of FiO_2_ in order to prevent hyperoxia is especially important in trauma patients with TBI undergoing PHEA. A significant proportion of the patients included in our cohort (n = 46, 22%) had confirmed TBI on imaging and of these 29 (63%) were exposed to severe hyperoxia. Previous evidence suggests that patients suffering ischaemic/reperfusion injuries, such as TBI, are especially sensitive to the adverse effects of hyperoxia. Menzel et al. demonstrated a substantial decrease in cerebral perfusion following hyperoxaemia in TBI patients, mediated via cerebral vasoconstriction, this mechanism may create ischemia and exacerbate injury [23]. Further, in a retrospective review of 1547 patients with severe TBI it was found that both exposure to hyperoxemia and hypoxemia within the first 24 h of hospitalisation was associated with worse short-term functional outcomes and higher mortality [20].

Pre-hospital critical care teams should not regard supplemental oxygen as a protective therapy for all trauma patients after PHEA. Especially when the risk of hypoxemia is deemed low (e.g. absence of chest injuries, no suspicion of TBI), early down-titration of FiO_2_ should be considered. Using pulse oximetry SpO_2_ values in the pre-hospital setting to accomplish this may prevent prolonged periods of hyperoxemia, and resultant deleterious effects.

## Limitations

Our study has several limitations inherent to the retrospective study design. First, we had to rely on the data as provided by the HEMS team. Although there were some missing data, overall data completeness was good due to the use of our electronic patient record with dedicated data entry fields for all patients. Further, we included patients whom received and ABGA within 60-min of arrival to hospital, and in-hospital oxygen therapy adjustments immediately after handover and before the ABGA may have confounded our findings. However, despite that FiO_2_ down-titrated after handover in 120/147 patients, the vast majority were found to have significant hyperoxemia in the ABGA. Whether this was the result of prolonged hyperoxemia in the pre-hospital setting, inadequate FiO_2_ down-titration in hospital before the ABGA was performed, or both, could not be established. However, it is likely that our findings reflect a general lack of awareness to prevent hyperoxemia in intubated trauma patients. It is important to mention that our findings are not extrapolatable to other (non-trauma) populations, who may have a different SpO_2_ target based on their presenting pathophysiology. Further, it should be mentioned that SpO_2_ can only be used to guide oxygen therapy when a reliable pulse oximetry trace is recorded, which often is not the case in patients with significant hypovolemic shock, and in patients with severe hypothermia. Finally, this study was aimed to investigate the incidence of hyperoxemia, and to determine patient factors that may help guide clinicians with pre-hospital oxygen titration in these patients. As we did not collect (patient centered) outcome data, we could not determine any associations between (the degree of) hyperoxemia and outcome.

## Conclusion

Trauma patients who have undergone PHEA often (61–81%) have a profound hyperoxemia within 60-min of arrival to hospital. In the pre-hospital setting, where ABGA are not readily available, a titrated approach to oxygen therapy should be considered to reduce the incidence of potentially harmful tissue hyperoxia.

## Data Availability

The datasets used and/or analysed during the current study are available from the corresponding author on reasonable request.

## Abbreviations

AAKSS: Air Ambulance Kent Surrey Sussex
ABGA: Arterial blood gas analysis
ApOx: Apnoeic Oxygenation
CNS: Central Nervous System
ED: Emergency Department
GCS: Glasgow Coma Score
HEMS: Helicopter Emergency Medical Service
IPPV: Intermittent Positive Pressure Ventilation
KSSAAT: Kent Surrey Sussex Air Ambulance Trust
MOI: Mechanism of Injury
MTC: Major Trauma Centre
NIHR: National Institute for Healthcare Research
OR: Odds Ratio
PHEA: Pre-hospital Emergency Anesthesia
ROS: Reactive oxygen species
RSI: Rapid Sequence Induction
RTCs: Road Traffic Collisions
SOP: Standard Operating Procedure
STGH: St George’s Hospital
SWLSTN: South West London and Surrey Trauma Network
TBI: Traumatic Brain Injury
